# Genomic and Biological Characterization of a New Cypovirus Isolated from *Dendrolimus punctatus*

**DOI:** 10.1371/journal.pone.0113201

**Published:** 2014-11-24

**Authors:** Yin Zhou, Tongcheng Qin, Yuzhou Xiao, Fujun Qin, Chengfeng Lei, Xiulian Sun

**Affiliations:** Key Laboratory of Agricultural and Environmental Microbiology, Wuhan Institute of Virology, Chinese Academy of Sciences, Wuhan, Hubei, China; Plymouth University, United Kingdom

## Abstract

A novel cypovirus (designated DpCPV-MC) was isolated from the pine moth *Dendrolimus punctatus* using serial *in vivo* cloning procedures. DpCPV-MC occurs in typical polyhedral occlusion bodies, containing a number of spherical virions. Laboratory bioassays indicated that the infectivity of DpCPV-MC against second-instar *Spodoptera exigua* larvae does not differ significantly from that of *Dendrolimus punctatus cypovirus 1*. Full-length amplification of the DpCPV-MC cDNAs identified 16 dsRNA genome segments. Each segment encodes one open reading frame with unique conserved terminal sequences at the 5′ and 3′ ends, which differ from those of all previously reported cypoviruses. On a phylogenetic tree based on the amino acid sequences of the polyhedrin of 19 cypovirus species, DpCPV-MC was closest to the type-4 cypoviruses. Homology searches showed that ten segments of DpCPV-MC (S1, S2, S3, S4, S5, S7, S8, S9, S12, and S13) encode putative CPV structural and nonstructural proteins, three segments (S6, S10 and S14) encode putative insect proteins or other viral proteins, and the other three segments (S11, S15, and S16) encode proteins that have no obvious sequence similarity to any known protein. Based on RNA secondary structures analysis, two segments of them (S11 and S16) were predicted to possibly transcript less efficiently than the other segments. We speculate that DpCPV-MC is composed of several genotypes. The ten CPV-related segments constantly exist in all genotypes, and one or two of the six CPV-unrelated segments co-exist with the ten CPV-related segments in one DpCPV-MC genotype, thus each virion contains no more than 12 segments. Based on our results and the literature, DpCPV-MC is a new cypovirus (*Cypovirus 22*, strain DpCPV-22).

## Introduction

Cypoviruses (CPVs), of the family *Reoviridae*, have been isolated from various insects in the Lepidoptera, Diptera, Hymenoptera and Coleoptera [1]. CPVs are segmented double-stranded RNA viruses that infect the midgut cells of insect larvae and replicate in the cytoplasm of insect cells. CPV virions are occluded in polyhedral occlusion bodies (OBs), which are formed of the polyhedrin protein encoded by the viral genome. The OBs dissolve in the alkaline environment of the insect midgut and the virions are released to initiate the infection process. The capsids of CPVs are single-layered and icosahedral in shape, with a diameter of 50–80 nm [1]. The morphological features of the CPV capsid have been investigated with cryoelectron microscopy combined with a three-dimensional reconstruction method [2], [3]. Atomic models have revealed that the CPV capsid is composed of three major structural proteins: the capsid shell protein, turret protein, and large protrusion protein [2]. Twelve transcriptase enzyme complexes are attached to the inner surface of the capsid shell [4].

The CPVs have so far been classified into 21 types based on the electrophoretic migration patterns of their dsRNA genome segments [5]. Several CPVs have been completely or partially sequenced, including *Bombyx mori cypovirus 1* (BmCPV-1) [6], [7], [8], [9], [10], [11], *Dendrolimus punctatus cypovirus 1* (DpCPV-1) [12], [13], and *Antheraea mylitta cypovirus 4* (AmCPV-4) [14], [15], [16], [17], [18], [19], [20]. The conserved terminal sequences of CPVs vary in different CPV types, e.g., the conserved terminal sequences of AmCPV-4 are 5′-AGTAAT…AGAGC-3′ [16]. The structural proteins and nonstructural proteins of AmCPV-4 have been characterized. The minor capsid protein and major capsid protein are encoded by genome segments 1 and 3, respectively [19], the RNA-dependent RNA polymerase is encoded by segment 2 [20], and polyhedrin is encoded by segment 10 [15]. Its other proteins function in RNA binding and replication, including p68, p61, p60, and NSP38, which are encoded by segments 6, 7, 8, and 9, respectively [14], [16], [17], [18]. There are usually 10 equimolar double-stranded RNA (dsRNA) segments in the genomes of CPVs, but some CPVs have an 11^th^ segment [15], [21].

In this study, a new CPV, designated DpCPV-MC, was isolated from the pine moth *Dendrolimus punctatus* and characterized. The complete sequences of the 16 dsRNA segments of DpCPV-MC were obtained with the full-length amplification of cDNAs (FLAC) technique. A further homology analysis of these segments was performed, and a phylogenetic analysis was conducted based on the amino acid sequences of the polyhedrin proteins of different CPVs. The results of this study suggest that DpCPV-MC is a new type of CPV composed of several genotypes.

## Materials and Methods

### Isolation, propagation, and purification of DpCPV-MC

Isolates of DpCPV-MC were originally collected from diseased *D. punctatus* larvae in Macheng, Hubei, China, in 2004. DpCPV-MC was propagated in the laboratory in fourth-instar *Spodoptera exigua* (Hubner) larvae as an alternative host [22]. The larvae were reared on a bean-flour-based artificial diet at 27±1°C [23]. The OB concentrations were determined with a hemocytometer under a light microscope. The original isolate was a mixture of DpCPV-1 and a novel electrophoretic type. DpCPV-MC was separated from the mixture with serial *in vivo* cloning procedures [24], [25]. Briefly, a low concentration (10^3^ OB/mL) of the isolated CPV mixture, which might cause about 5% mortality (LC_5_), was added to the artificial diet and used to inoculate second-instar larvae of *S. exigua*. The OBs were collected from individual larvae and used to inoculate second-instar *S. exigua* at a low concentration (LC_5_). Four rounds of this *in vivo* cloning procedure were performed until no segments of DpCPV-1 were present in the electrophoretic profiles.

### Insecticidal bioassays

Second-instar larvae of *S. exigua* were starved overnight and fed with an artificial diet contaminated with 5 µl of different concentrations (10^2^–10^7^ OB/mL) of CPVs. Purified DpCPV-1 was assayed in parallel, as the control. Forty-eight larvae were used for each viral treatment. Larvae fed on the artificial diet and treated with only distilled water were also used as a control. The larvae were reared at 27±1°C and 60%–70% humidity. Larval mortality was recorded daily until all the tested larvae had either died or pupated. The bioassays were performed in duplicate. The LC_50_ and 95% confidence interval (CI) of each virus were determined with a probit analysis using the computer package SPSS 13.0 (SPSS Inc., Chicago, IL, USA) [26]. The data from two replicates were pooled to calculate the final LC_50_ values if the two replicates did not differ significantly. The LC_50_ values for DpCPV-1 and DpCPV-MC were compared with a standard lethal dose ratio comparison [27].

### Purification of DpCPV-MC virions and electron microscopy

The purified OBs were fixed with 5% glutaraldehyde and 2% sucrose (pH 7.3) for 5 h, and with 1% osmic acid for 1 h, dehydrated in a graded series of ethanol, embedded in 618 epoxy resin, and cut into ultrathin sections. The samples were observed with a Hitachi H-7000FA transmission electron microscope (TEM) at an acceleration voltage of 75 kV. The purified OBs were lysed for 5 min in 0.2 M Na_2_CO_3_·NaHCO_3_ (pH 10.8) at 4°C, and any undissociated polyhedra were removed by centrifugation (3,000×g, 10 min). The supernatant was purified by linear 20%–60% (w/v) sucrose gradient centrifugation at 60,000×g for 2 h at 4°C. The milky band of virions was collected, diluted with PBS (pH 7.4), and centrifuged (90,000×g, 70 min, 4°C) to remove the sucrose. The virions were observed with a Hitachi H-7000FA TEM at an acceleration voltage of 75 kV.

### Preparation of viral dsRNA and electrophoretic analysis

TRIzol Reagent (Invitrogen, Carlsbad, CA, USA) was used to extract the genomic dsRNA from the purified OBs and purified virions of DpCPV-MC. The isolated dsRNA was analyzed on 1% agarose gel and then stored at −70°C.

### cDNA synthesis and cloning

The full-length amplification of cDNAs (FLAC) strategy [28], [29] was used to obtain the entire sequences of the genomic segments of DpCPV-MC. First, a 35-base oligonucleotide ‘anchor-primer’ (5′-p-GACCTCTGAGGATTCTAAAC/iSp9/TCCAGTTTAGAATCC-OH-3′) was synthesized, with a C9 (phosphoramidite) spacer (iSp9) between the two complementary halves and a phosphorylated 5′ terminus (Sangon, Shanghai, China), and ligated to both ends of the dsRNA segments. The ligation reactions were performed with T4 RNA ligase (New England Biolabs, Hitchin, UK) [28]. After the reaction, the dsRNA with the anchor-primer was extracted with the Zymoclean Gel RNA Recovery Kit (Zymo Research, Orange, CA, USA). The first-strand cDNA of each genome segment was reverse transcribed with AMV reverse transcriptase (10 U/µl, Promega, Madison, WI, USA), and used for polymerase chain reaction (PCR). Amplification was performed with primer 5-15-1, 5′-GAGGGATCCAGTTTAGAATCCTCAGAGGTC-3′ containing a *Bam*HI restriction site (underlined). The PCR products were purified with a gel extraction kit (Omega Bio-Tek, Norcross, GA, USA), and cloned into pMD18-T (TaKaRa, Tokyo, Japan). Competent *Escherichia coli* DH5α cells were transformed with the ligation products. The positive plasmids were sequenced on both strands using the M13 universal primers (Sangon). At least five clones were sequenced for each reverse-transcribed PCR product.

### Bioinformatic analysis

The open reading frame (ORF) of each dsRNA segment of DpCPV-MC was identified by the National Center for Biotechnology Information (NCBI) ORF Finder online service, http://www.ncbi.nlm.nih.gov/gorf/gorf.html, and translated into the amino acid sequence for further analysis. Homology searches were performed with the BLASTp program at NCBI online services, http://blast.ncbi.nlm.nih.gov; only homologous proteins with an E-value <1×10^−5^ were listed and compared with the predicted proteins from DpCPV-MC. Information on the different types of CPV was obtained from the website: http://www.reoviridae.org/dsRNA_virus_proteins/CPV-RNA-Termin.htm.

The amino acid sequences of the polyhedrin proteins of 18 species of CPV were retrieved from the NCBI GenBank database. The amino acid sequence of DpCPV-MC polyhedrin was predicted by the NCBI ORF Finder online service. A phylogenetic tree of the polyhedrin proteins of 19 CPV species was constructed with the ClustalX2 and Molecular Evolutionary Genetics Analysis (Mega) 5.2 software using the neighbor-joining method with the default parameters [30]. The secondary structures of the 16 dsRNAs of DpCPV-MC were predicted with the online program RNAfold [31].

### *S. exigua* genomic DNA extraction

Twenty second-instar (L2) *S. exigua* larvae were collected in pestle, flash frozen by liquid nitrogen, and grinded into powders. Appropriate volume of extracting buffer (10 mM Tris-Cl, 100 mM EDTA, 20 µg/ml RNase A, 0.5% SDS) was added into the powers and mixed well. After incubating 1 h at 37°C, proteinase K was added at a final concentration of 100 µg/ml and mixed well. After incubating 3 h at 50°C, the genomic DNA of *S. exigua* larvae was purified via Tris-phenol extraction and ethanol precipitation. The genomic DNA was dissolved in ddH_2_O, and quantified with NanoDrop 2000 Spectrophotometer (Thermo Fisher Scientific, Co., USA) for southern blot hybridization.

### Northern and southern blot analyses

Northern blot hybridization was performed to verify if the six segments (S6, S10, S11, S14, S15 and S16), which are not related to those of any existed CPV sequence, are present in the genome of DpCPV-MC. A southern blot hybridization was performed to detect if these segments could hybrid with the genome of the host larvae. The probes for both northern and southern blot hybridization were generated by PCR with the primers shown in Table 1 and a random primer labeled with digoxigenin-11–dUTP using the DIG High Prime DNA labelling and detection starter kit I (Roche Diagnostics, Mannheim, Germany).

**Table 1:** Primers used in this study.

Target segment	Primer name	Primer sequence
S6	S6-up	ACGGCACTCCTGTTTTAT
	S6-dw	TTTAACCTTTCCTCGGTC
S10	S10-up	TCACTCCTATGCCACAAC
	S10-dw	CTAAATAAACCGCAATCC
S11	S11-up	AAACGCAAGCGATAAGAG
	S11-dw	AAGGGAATGGCAAATACAG
S14	S14-up	ATGGACGCCACTCTGAAT
	S14-dw	ATGGGCTCGATTGTTAGA
S15	S15-up	TCGGTTCGAGATGTTGGC
	S15-dw	CATGTTTGGTCTGGCGTGT
S16	S16-up	TGACCGCACGAGCAAGTT
	S16-dw	CGTCTCAGTTCACGCTGTAGTT
PBP3	PBP3-up	AGCATTTTCGTCGTTTGG
	PBP3-dw	GAGGGGTCTTACCGCTGC

For northern blot hybridization, the genomic dsRNA segments of DpCPV-MC were extracted from purified virions, and separated on 1% agarose gel and transferred onto nylon membrane (Roche Diagnostics, Mannheim, Germany) by capillary transfer procedure in 20× SSC buffer. The membrane was separatedly hybridized with digoxigenin (DIG)-labeled DNA probes specific for segments S6, S10, S11, S14, S15, and S16.

For southern blot hybridization, a pair of primers was designed to amplify the pheromone-binding protein 3 (PBP3) gene of *S. exigua* as a positive control of *S. exigua* genome. To perform southern blot hybridization, genomic DNA of *S. exigua* larvae was digested with *Bam*HI or *Xho*I. The digested genomic DNA was separated by electrophoresis and transferred onto the nylon membrane by capillary transfer procedure (Roche). For the membrane hybridized with probe mixture of six DpCPV-MC dsRNA segments (S6, S10, S11, S14, S15 and S16), pMD18-T vectors containing full-length sequence of the six DpCPV-MC segments were used for positive control.

The detection for northern and southern blot hybridization was performed using a DIG-high prime DNA labeling and detection starter kit I (Roche), according to the manufacturer's instructions. Each reaction had been repeated at least three times.

## Results

### Electrophoretic analysis, viral morphology, and bioassays

**Figure 1:**
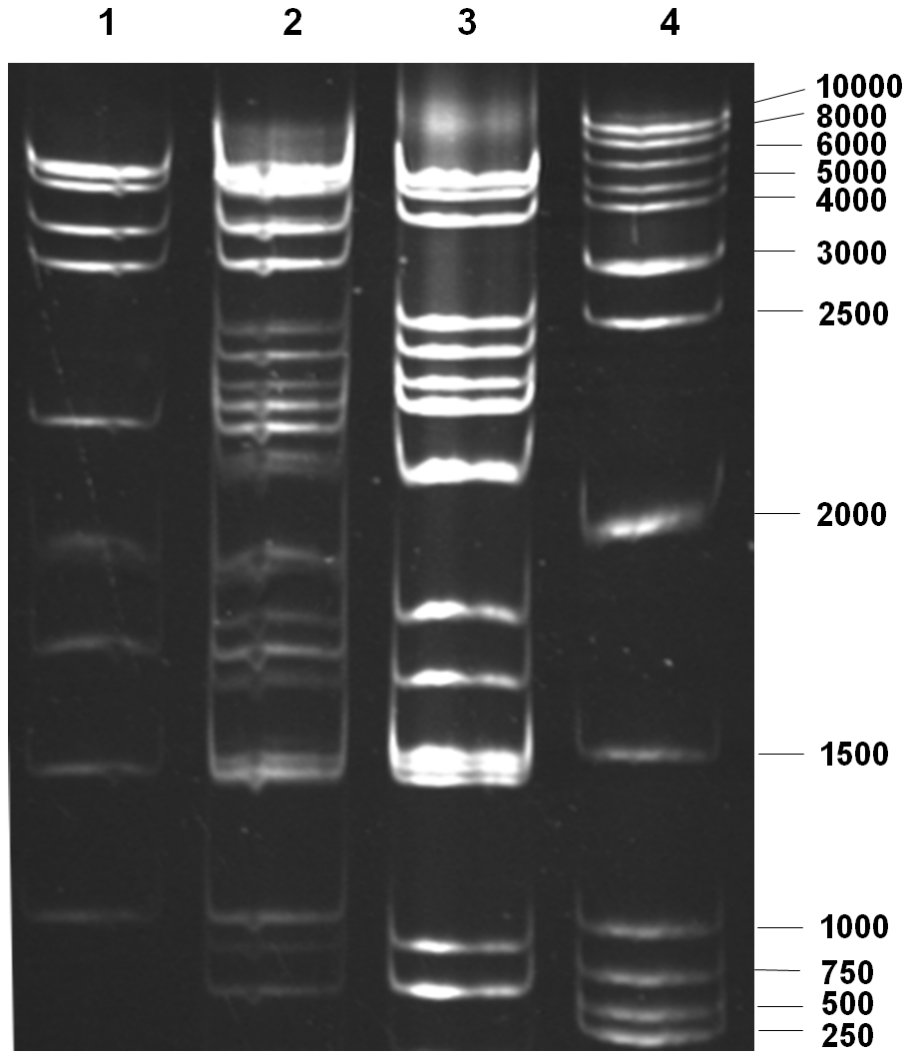
Electrophoretic analysis of DpCPV-MC before and after the *in vivo* cloning procedure. Electrophoresis was performed in 1× TAE (40 mM Tris–acetate, 1 mM EDTA, pH 7.8) at a constant voltage of 50 V for 4 h on a 13% polyacrylamide gel stained with ethidium bromide. Lane 1: DpCPV-1; lane 2: CPV mixture originally collected from *D. punctatus*; lane 3: DpCPV-MC; lane 4: 1-kb DNA ladder (Promega).

**Figure 2:**
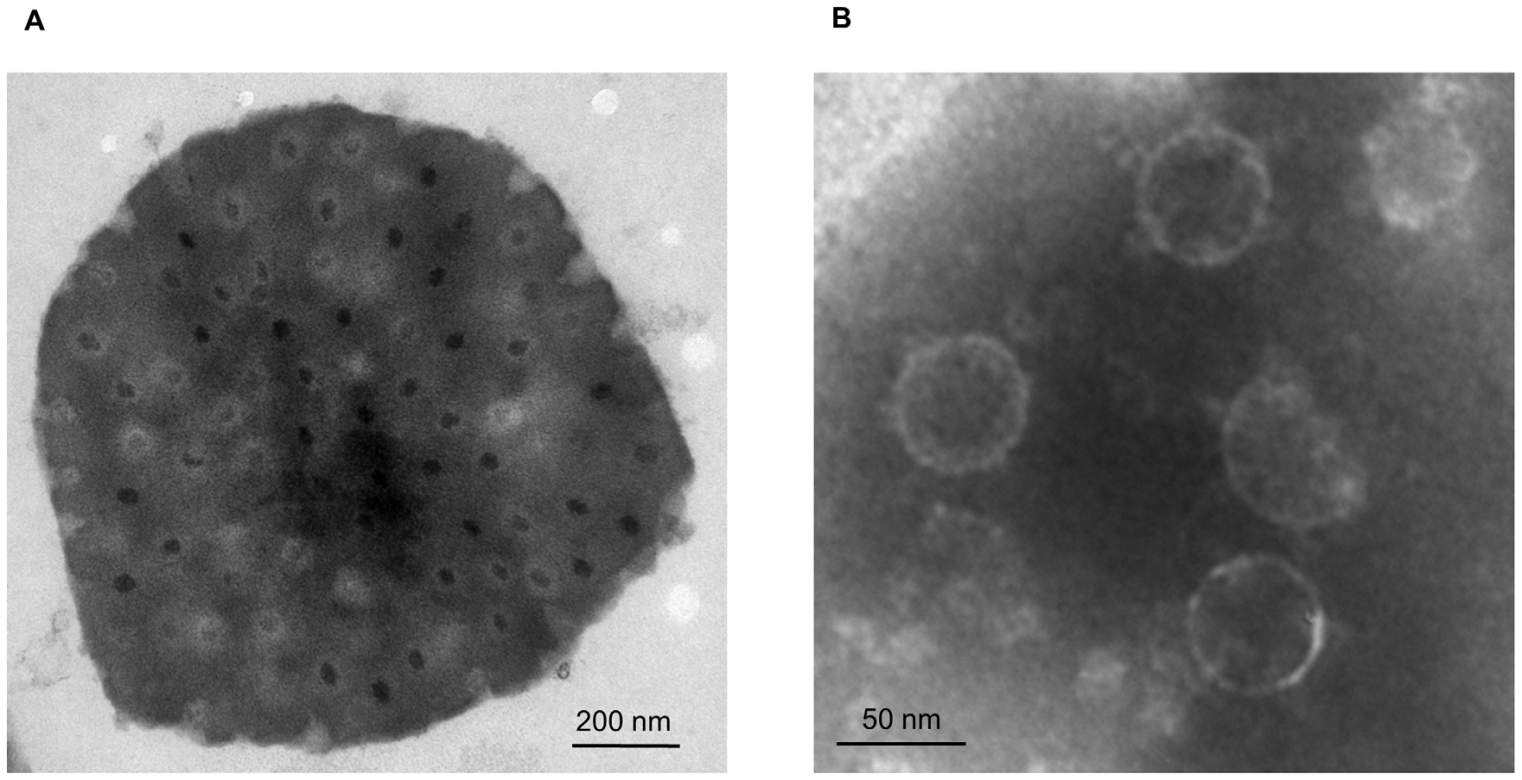
Electron micrographs of OBs of DpCPV-MC. (A) Transmission electron micrograph of ultrathin sections of OBs of DpCPV-MC. (B) Transmission electron micrograph of purified virions of DpCPV-MC.

When DpCPV-MC was originally isolated from pine moth larvae, it was present in a mixture with DpCPV-1. We attempted to separate the new CPV from the mixture using serial *in vivo* cloning procedures. After four rounds of *in vivo* cloning, DpCPV-1 was successfully removed from the mixture, and a new electrophoretic type consisting of 15 bands was isolated (Figure 1). This isolate contained no DpCPV-1 genomic segments and was designated “DpCPV-MC”. A number of spherical viral particles were observed in ultrathin sections of DpCPV OBs (Figure 2A), with a diameter of approximately 50 nm (Figure 2B). Bioassays of second-instar *S. exigua* larvae showed that the infectivity of DpCPV-MC did not differ significantly from that of DpCPV-1 (Table 2).

**Table 2:** Median lethal concentrations (LC_50_) of DpCPV-MC and DpCPV-1 against second-instar *S. exigua* larvae.

Viruses	LC_50_ (×10^4^ OBs/mL, with95% CI)	Slope ± SE	Potency ratio a (95% CI)
DpCPV-1	4.9 (0.8, 16.2)	0.790	-
DpCPV-MC	1.1 (0.2, 1.51)	0.448	0.26 (0.03, 3.04)

a Potency ratio was calculated by dividing the LC_50_ of DpCPV-MC by that of DpCPV-1. Significant difference was based on whether the 95% confidence interval (CI) of the potency ratio included the value 1.0 [47].

### Determination of DpCPV-MC dsRNA segment sequences

**Figure 3:**
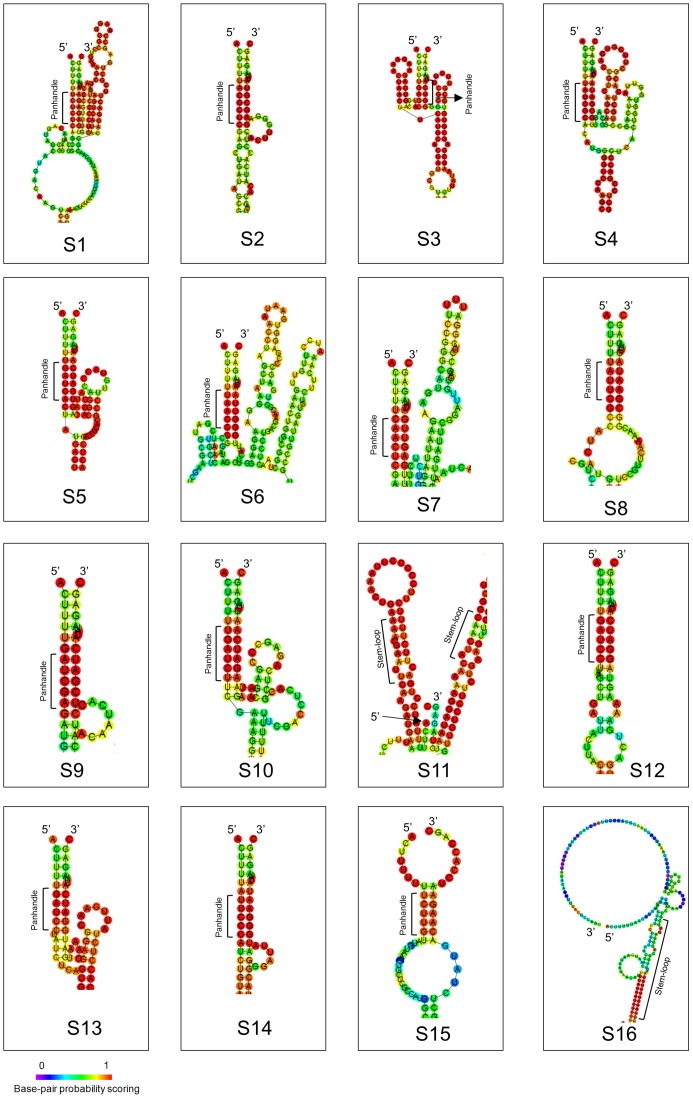
Folding of the 5′ and 3′ NCRs of the DpCPV-MC genome segments. The RNAfold program was used to predict the secondary structures formed by the 16 DpCPV-MC genome segments. The panhandle structure was formed by base-pairing between the 5′ and 3′ ends, and the stem–loop structure was formed by either the 5′ or 3′ terminal sequence. The panhandle structure and the stem–loop structure are marked with long brackets.

In total, 16 genome segments were identified from the sequencing results after the FLAC procedure (Table 3). Their GenBank accession numbers are shown in Table 3. The lengths of the 16 segments range from 4051 to 783 nucleotides (nts). All the segments have the conserved terminal sequences 5′-ACUUUU…UAGAGC-3′, except S15 and S16, in each of which the 3′ terminal sequences is CCAGC-3′. The conserved terminal sequences differ from those of other previously reported CPV types [32]. The folding predicted with RNAfold showed that the 5′ and 3′ noncoding regions (NCRs) of 14 segments (S1, S2, S3, S4, S5, S6, S7, S8, S9, S10, S12, S13, S14, and S15) form typical panhandle structures by base-pairing between their 5′ and 3′ ends. No obvious panhandle structures were predicted to form between the 5′ and 3′ NCRs of the other two genome segments (S11 and S16), but potential stem–loop structures were predicted in either the 5′-terminal or 3′-terminal sequence (Figure 3).

**Table 3:** Properties of the dsRNA segments of DpCPV-MC.

Segment number	Accession number	Segment size (nt)	ORF position (nt)	Analysis of the predicted protein				
				Protein size (aa)	Putative Function	Most homologous species	Query coverage	Amino acid identity
S1	KJ191104	4051	33–3971	1312	minor capsid protein	*Heliothis armigera* cypovirus 5	94%	29%
S2	KJ191105	3713	125–3622	1165	major capsid protein	*Antheraea mylitta* cypovirus 4	98%	34%
S3	KJ191106	3650	35–3607	1190	RNA-dependent RNA polymerase	*Antheraea mylitta* cypovirus 4	87%	45%
S4	KJ191107	3382	15–3329	1104	mRNA guanylyltransferase and methyltransferase	*Choristoneura occidentalis* cypovirus 16	99%	25%
S5	KJ191108	2351	329–2251	640	guanylyltransferase	*Antheraea mylitta* cypovirus 4	70%	22%
S6	KJ191109	2203	16–2004	662	unknown	*Harpegnathos saltator*	77%	32%
S7	KJ191110	2005	29–1921	630	help RNA replication and transcription	*Antheraea mylitta* cypovirus 4	99%	28%
S8	KJ191111	1879	17–1720	567	help RNA replication and transcription	*Antheraea mylitta* cypovirus 4	32%	21%
S9	KJ191112	1695	14–1633	539	nucleotide biosynthesis and RNA replication	*Antheraea mylitta* cypovirus 4	53%	29%
S10	KJ191113	1431	95–1327	410	poly (ADP-ribose) glycohydrolase	*Naegleria gruberi*	46%	35%
S11	KJ191114	1339	53–1159	368	/	No significant similarity found.	/	/
S12	KJ191115	1160	32–1093	353	RNA binding protein NSP38	*Antheraea mylitta* cypovirus 4	91%	21%
S13	KJ191116	1155	30–797	255	polyhedrin	*Antheraea mylitta* cypovirus 4	98%	51%
S14	KJ191117	1143	90–956	289	acetyltransferase	*Pieris rapae* granulovirus	68%	40%
S15	KJ191118	813	30–725	231	/	No significant similarity found.	/	/
S16	KJ191119	783	38–706	222	/	No significant similarity found.	/	/

### Homology analysis of DpCPV-MC amino acid sequences

The basic properties of the protein encoded by each dsRNA segment of DpCPV-MC and the proteins most homologous to them (with the highest scores) in the NCBI GenBank database are listed in Table 3. The putative functions of the DpCPV-MC-encoded proteins, extrapolated from those of their homologous proteins, are also listed.

The largest segment of DpCPV-MC (S1) has a long ORF of 3939 nts, encoding a protein of 1312 amino acids with a molecular mass of ∼148 kDa. The deduced amino acid sequence encoded by S1 shows greatest homology to P2 of *Heliothis armigera cypovirus 5* (HaCPV-5), encoded by its genomic segment 2 (GenBank accession number: YP_001883322). It is also similar (26%) to the minor capsid protein of AmCPV-4, encoded by its genomic segment 1 [19]. The minor capsid protein of AmCPV-4 associates with the major capsid protein to maintain its stability [19].

The second segment (S2) is predicted to contain a single large ORF between nt 125 and nt 3622, encoding a predicted protein of 1165 amino acids with a calculated molecular mass of ∼132 kDa. The predicted protein shares 34% amino acid sequence identity with the major capsid protein of AmCPV-4, encoded by its segment 3 [19]. It also shares 24% amino acid identity with VP1 of DpCPV-1 (GenBank accession number: AAN84544), 22% with VP1 of *Heliothis armigera cypovirus 14* (GenBank accession number: ABD57841), and 22% with VP3 of *Aedes pseudoscutellaris reovirus* (GenBank accession number: YP_443937).

Segment 3 of DpCPV-MC contains a single large ORF between nt 35 and nt 3607, encoding a predicted protein with a molecular mass of ∼136 kDa that shares 45% amino acid identity with the RNA-dependent RNA polymerase (RdRp) of AmCPV-4 [20]. It also shares 32% amino acid identity with RdRp of *Choristoneura occidentalis cypovirus 16* (CoCPV-16; GenBank accession number: ACA53380), and 21%–28% amino acid identity with RdRps from other CPVs or other members of the family *Reoviridae*, such as *Aedes pseudoscutellaris reovirus* (GenBank accession number: YP_443936) and *Rice ragged stunt virus* (GenBank accession number: AEC32904).

Segment 4 of DpCPV-MC contains a single large ORF between nt 15 and nt 3329, encoding a predicted protein of 1104 amino acid with a molecular mass of ∼126 kDa. This protein shares 25% amino acid identity with an unknown protein of CoCPV-16, encoded by its segment 4 (GenBank accession number: ACA53382), and 22% amino acid identity with VP3 of BmCPV-1 (GenBank accession number: BAB17720) and the corresponding protein in DpCPV-1 (GenBank accession number: AAN17823). VP3 of BmCPV-1 functions as an mRNA guanylyltransferase and methyltransferase [33].

Segment 5 of DpCPV-MC contains an ORF between nt 329 and nt 2251 that encodes a predicted protein with a molecular mass of ∼73 kDa. The alignment showed 70% query cover and 22% amino acid identity between the deduced protein of S5 and the guanylyltransferase of AmCPV-4 encoded by its segment 5 (GenBank accession number: AGO04425).

Segment 6 of DpCPV-MC contains an ORF between nt 16 and nt 2004 that encodes a predicted protein with a molecular mass of ∼75 kDa. The deduced amino acid sequence shows 32% identity with an unknown protein of *Harpegnathos saltator* (GenBank accession number: EFN83910), and 22%–44% amino acid identity with unknown proteins in other insect species, including *B. mori* (GenBank accession number: XP_004922867). It also shares 20% amino acid identity with glucose-6-phosphate isomerase of the bacteria *Prevotella salivae* (GenBank accession number: ERK01800).

The predicted viral protein encoded by S7 of DpCPV-MC shares 28% amino acid identity with P68 of AmCPV-4, with an E-value of 2×10^−58^. The predicted protein encoded by S8 of DpCPV-MC shares 21% amino acid identity with P61 of AmCPV-4 [16]. P68 binds and hydrolyses ATP, and P61 binds single-stranded viral RNA. These two proteins are thought to participate in viral RNA replication and transcription [16], [18].

The predicted protein encoded by S9 of DpCPV-MC shares 29% amino acid identity with P60 of AmCPV-4 [17], which might be involved in nucleotide biosynthesis and viral RNA replication. It also shares 22% amino acid identity with the predicted protein encoded by CoCPV-16 segment 7 (GenBank accession number: ABW87641), 33% amino acid identity (33% query cover) with VP5 of HaCPV-5, encoded by its segment 8 (GenBank accession number: ABA10825), and 19% amino acid identity (33% query cover) with VP5 of *Operophtera brumata cypovirus 18*, encoded by its segment 7 [34].

Segment 10 of DpCPV-MC is predicted to encode a protein composed of 410 amino acids with a molecular mass of ∼46 kDa. Its C-terminal subunit (amino acids 207–406) shows 33%–35% amino acid identity with poly(ADP-ribose) glycohydrolase of *Naegleria gruberi* (GenBank accession number: EFC39385) and other insect species.

Segment 12 of DpCPV-MC contains a single large ORF encoding a predicted protein of 353 amino acids with a molecular mass of ∼39 kDa, with 21% identity to a nonstructural RNA-binding protein (NSP38) of AmCPV-4 [35].

**Figure 4:**
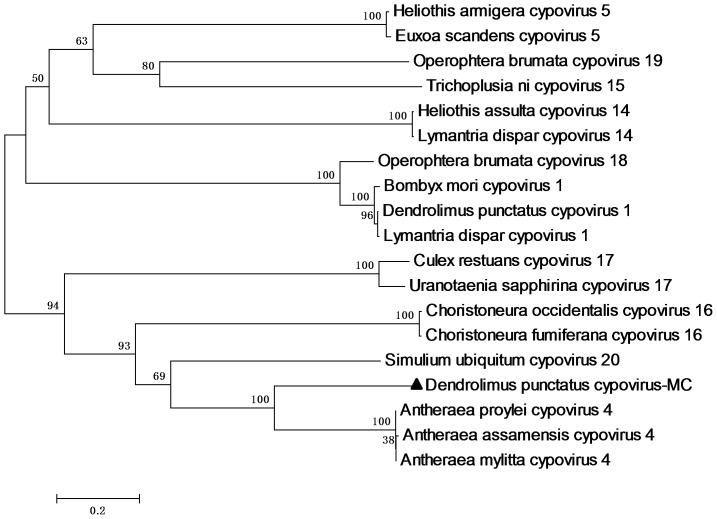
Neighbor-joining tree constructed from polyhedrin amino acid sequences of representative CPVs. Sequences were aligned with the multiple sequence alignment program ClustalX2. The neighbor-joining method was used to construct the phylogenetic tree of the derived polyhedrin protein sequences with the MEGA software version 5.2. The black triangle shows the position of DpCPV-MC. Bootstrap percentage values are indicated on the left. All reference sequences used for the construction of this tree were retrieved from GenBank with their corresponding accession numbers: *Antheraea assamensis cypovirus 4*, AY212275; *Antheraea mylitta cypovirus 4*, AY212273; *Antheraea proylei cypovirus 4*, AY212276; *Dendrolimus punctatus cypovirus 1*, AY204879; *Bombyx mori cypovirus 1*, D37770; *Culex restuans cypovirus 17*, DQ212785; *Choristoneura occidentalis cypovirus 16*, EU201043; *Euxoa scandens cypovirus 5*, J04338; *Heliothis assulta cypovirus 14*, DQ077914; *Heliothis armigera cypovirus 5*, DQ077912; *Lymantria dispar cypovirus 1*, AF389471; *Operophtera brumata cypovirus 18*, DQ192250; *Operophtera brumata cypovirus 19*, DQ192254; *Simulium ubiquitum cypovirus 20*, DQ834386; *Trichoplusia ni cypovirus 15*, NC_002565; *Uranotaenia sapphirina cypovirus 17*, AY876384; *Lymantria dispar cypovirus 14*, AF389461; *Choristoneura fumiferana cypovirus 16*, U95954.

The predicted protein encoded by S13 of DpCPV-MC is most closely related to the polyhedrin of AmCPV-4 (98% query cover, 51% identity) [15]. It also shares 27%–32% amino acid identity with the polyhedrin proteins of *Simulium ubiquitum cypovirus 20* [5] and CoCPV-16 (GenBank accession number: ABW87644). Because the polyhedrin proteins of all CPVs are highly conserved, a neighbor-joining phylogenetic tree was constructed with the amino acid sequences of the polyhedrin proteins of 19 CPV species (Figure 4). The results demonstrate that the 19 CPV species cluster in two clades. DpCPV-MC clusters with AmCPV-4, *Antheraea assamensis cypovirus 4* (AaCPV-4), and *Antheraea proylei cypovirus* 4, whereas DpCPV-1 and BmCPV-1 occur in the other clade.

The predicted protein encoded by S14 of DpCPV-MC shows 40% amino acid identity with the acetyltransferase of *Pieris rapae granulovirus* (GenBank accession number: ACZ63540), and 35%–40% amino acid identity with unknown proteins from *Agrotis segetum granulovirus* (GenBank accession number: YP_006287), *Choristoneura occidentalis granulovirus* (GenBank accession number: ABC61182), and other granuloviruses.

The S11, S15, and S16 of DpCPV-MC contain single ORFs of 1107 nt, 696 nt and 669 nt in length, respectively, and have no obvious sequence similarity to any known protein (BLAST, E-values > 0.01).

### Six dsRNA segments were present in purified virions of DpCPV-MC

**Figure 5:**
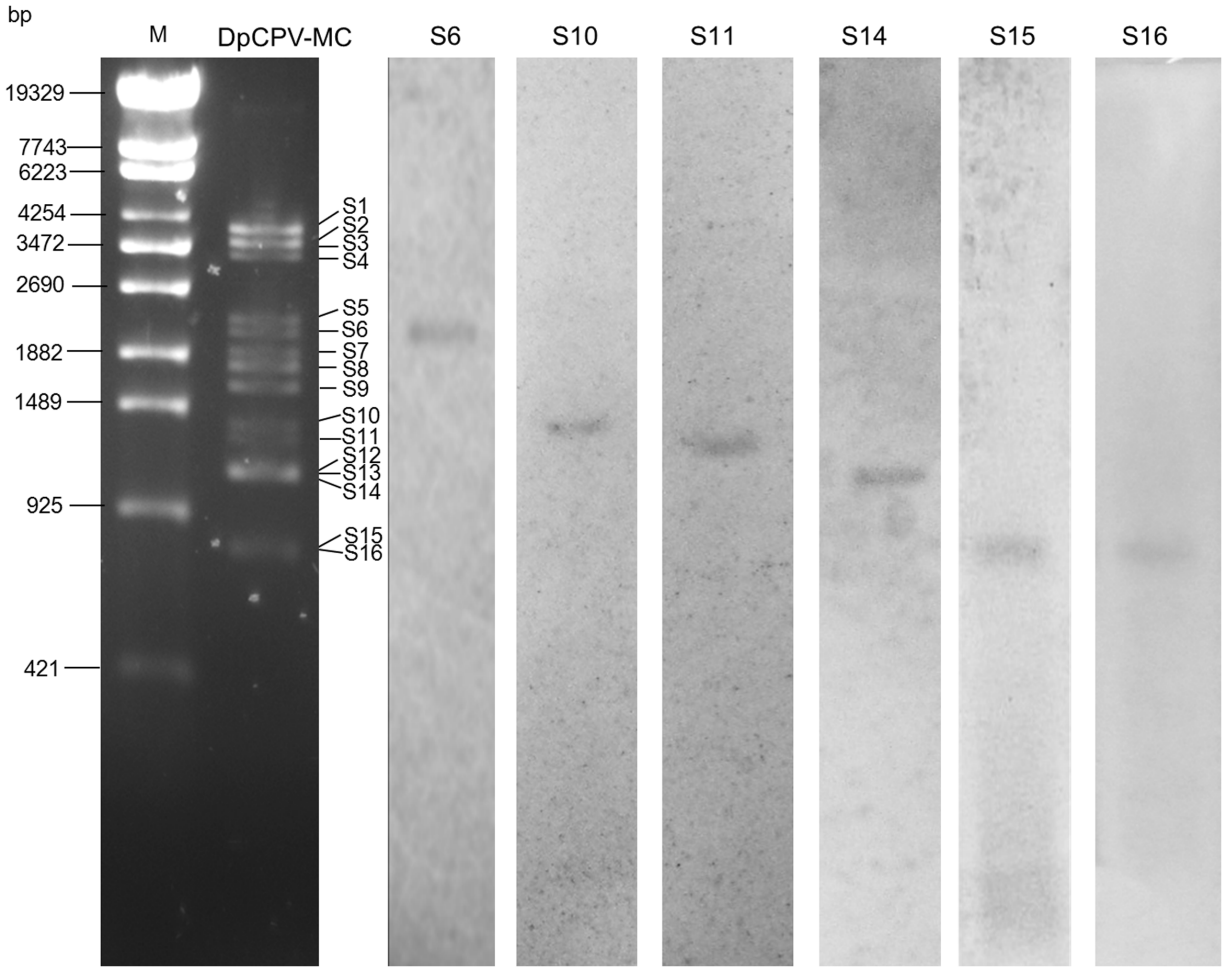
Northern blot analysis of six dsRNA segments of DpCPV-MC. Total genomic dsRNA of DpCPV-MC was separated on 1% agarose gel, transferred to nylon membrane, and hybridized with the corresponding DIG-labeled probes derived from each of the six segment sequences. The left part is agar gel stained with ethidium bromide. M, phage λ DNA digested with *Eco*T14 I. The right six lanes are northern blot results.

The amino acid sequences of the proteins predicted from six dsRNA genome segments (S6, S10, S11, S14, S15, and S16) were not similar to those of any CPV protein. In order to exclude the possibility of false positives caused by the FLAC procedure, and the possibility that some dsRNA segments are not present in virions, but embedded in OBs, northern blot hybridization was performed with RNA extracted from purified virions of DpCPV-MC. The results showed that the six dsRNA segments were detected by their corresponding segment-specific probes (Figure 5).

### Six dsRNA segments were not acquired from the host larvae

**Figure 6:**
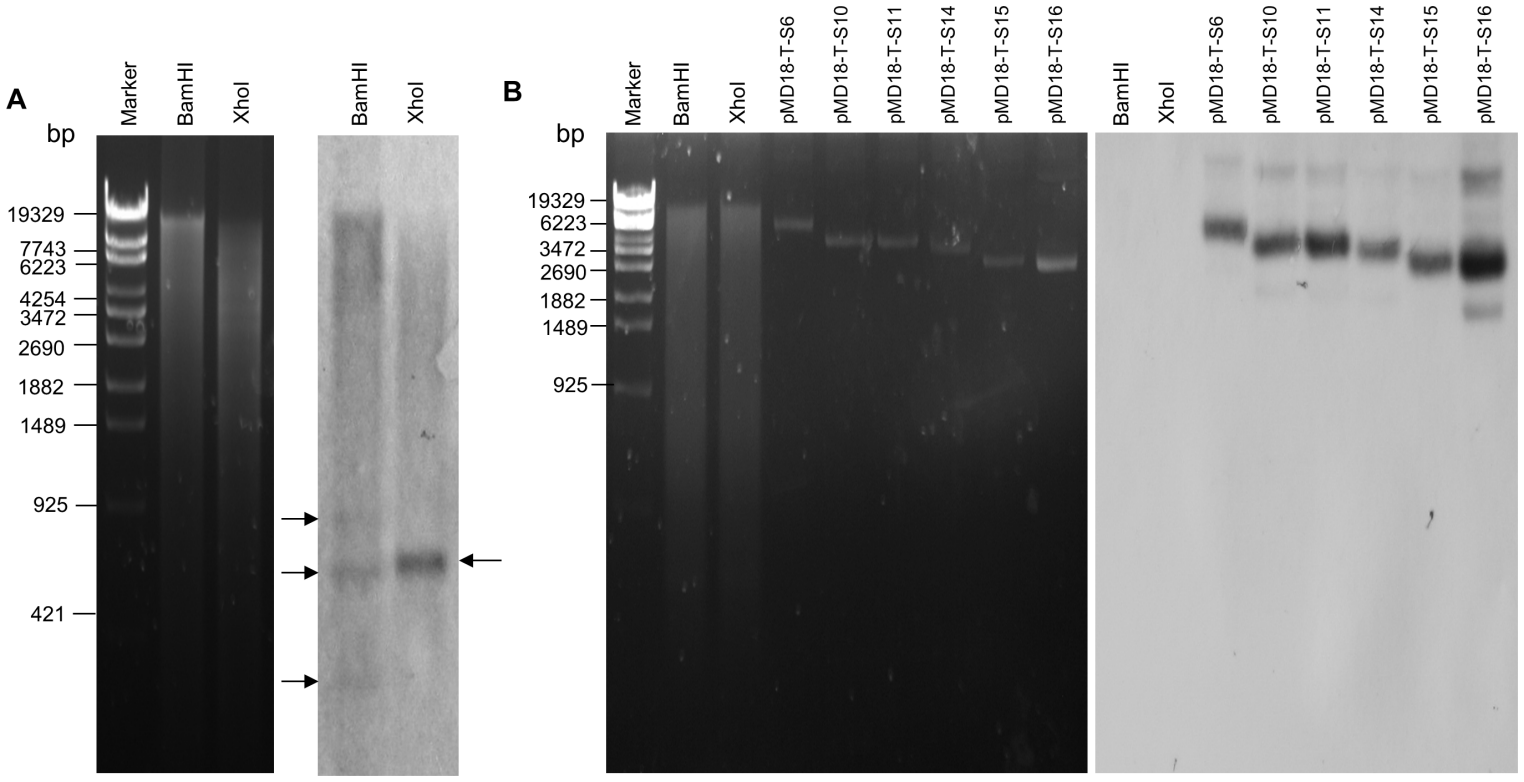
Southern blot analysis of genomic DNA of *S. exigua* larvae. A. Electrophoresis (left) and southern blot (right) of restriction endonuclease-digested genomic DNA of *S. exigua* larvae, detected by pheromone-binding protein 3 (PBP3) probe. Marker, phage λ DNA digested with *Eco*T14 I. XhoI, *Xho*I-digested genomic DNA of *S. exigua* larvae (2 µg). BamHI, *Bam*HI-digested genomic DNA of *S. exigua* larvae (2 µg). The arrows indicate hybridization bands in genomic DNA of *S. exigua* larvae. B. Electrophoresis (left) and southern blot (right) of restriction endonuclease-digested genomic DNA of *S. exigua* larvae, detected by probe mixture of six DpCPV-MC dsRNA segments (S6, S10, S11, S14, S15 and S16). The six plasmids containing full-length sequence of six segments was used as positive control. Marker, phage λ DNA digested with *Eco*T14 I. XhoI, *Xho*I-digested genomic DNA of *S. exigua* larvae (2 µg). BamHI, *Bam*HI-digested genomic DNA of *S. exigua* larvae (2 µg). pMD18-T-S6, pMD18-T vector containing full-length sequence of S6 (2 ng). pMD18-T-S10, pMD18-T vector containing full-length sequence of S10 (2 ng). pMD18-T-S11, pMD18-T vector containing full-length sequence of S11 (2 ng). pMD18-T-S14, pMD18-T vector containing full-length sequence of S14 (2 ng). pMD18-T-S15, pMD18-T vector containing full-length sequence of S15 (2 ng). pMD18-T-S16, pMD18-T vector containing full-length sequence of S16 (2 ng).

Southern blot hybridization analysis was performed to exclude the possibility that some DpCPV-MC dsRNA segments were RNA derivatives of the host that were caught by CPV replication machinery. The results showed that three bands (estimated sizes of 850 bp, 650 bp and 200 bp) were detected by the PBP3-specific probe in the lane of *Bam*HI-digested *S. exigua* genomic DNA, and one band (estimated size of 700 bp) was also detected by the PBP3-specific probe in the lane of *Xho*I-digested *S. exigua* genomic DNA, but no band could be detected by the probe mixture of six DpCPV-MC dsRNA segments (S6, S10, S11, S14, S15 and S16) in the lanes of restriction enzyme-digested *S. exigua* genomic DNA, it suggested that the six segments are not acquired from *S. exigua* larvae (Figure 6).

## Discussion

In this study, we identified a novel CPV (DpCPV-MC) from *D. punctatus* larvae with genomic segments containing unique conserved terminal sequences and with unique electrophoretic migration patterns. The conserved terminal sequences of the dsRNA segments in CPVs are important for RNA replication and packaging [36], and the conserved terminal sequences of the CPVs are one of the elements that distinguish the different CPV species [32]. The novel conserved terminal sequences of DpCPV-MC imply that it can be classified as a new type of CPV.

DpCPV-MC and DpCPV-1 were isolated from the same host insect, but they have different conserved terminal sequences at both their 5′ and 3′ ends. A phylogenetic analysis based on the amino acid sequences of 19 CPV polyhedrin proteins showed that DpCPV-MC and DpCPV-1 cluster in two independent clades, and DpCPV-MC is closest to the type-4 cypoviruses (Figure 4). Similarly, two types of CPVs, *Operophtera brumata cypovirus 18* (OpbuCPV-18) and *Operophtera brumata cypovirus 19* (OpbuCPV-19), have been isolated from *Operophtera brumata*. OpbuCPV-18 is closely related to DpCPV-1, whereas OpbuCPV-19 is closer to *Trichoplusia ni cypovirus 15* [*37*]. This implies that different CPVs isolated in the same host could have different evolutionary origins. Since many CPVs have a broad host range consisted by different lepidopteran insect pests [38], it is possible that *D. punctatus* may not even be the primary host of DpCPV-MC.

DpCPV-MC contains 16 genome segments. The predicted proteins encoded by 10 genome segments (S1, S2, S3, S4, S5, S7, S8, S9, S12, and S13) share some degree of amino acid identity with proteins of other CPVs, including all the structural and nonstructural proteins reported in other CPVs, e.g., major capsid proteins, minor capsid proteins, RdRps, polyhedrins, and RNA-binding proteins. The other six genome segments (S6, S10, S11, S14, S15, and S16) share no amino acid identity with any CPV protein, but their presence in RNA extracted from purified virions of DpCPV-MC was confirmed by northern blot (Figure 5). The result verified that all of the 16 segments are present in the genome of DpCPV-MC, and it also excluded the possibility that some dsRNA segments are embedded in polyhedra, but not present in virions.

Of the six segments that are unrelated to any other CPV protein, three (S6, S10, and S14) are related to an insect protein or another viral protein. The predicted protein encoded by S14 of DpCPV-MC shares 35%–40% amino acid identity with an unknown protein in four species of *Granulovirus*. Horizontal transfers were known to occur between baculoviruses [39], there is also an example that horizontal transfer between two different retrovirus lineages (mouse mammary tumor virus relatives and nonprimate lentiviruses) [40]. It has been proposed that when a CPV and a granulovirus coexist in the midgut of a common host larva, genes from the insect or granulovirus can be transferred to the CPV genome. The predicted protein encoded by S6 of DpCPV-MC is related to a protein from *H. saltator* and other insect species. The predicted protein encoded by DpCPV-MC S10 shares 35% amino acid identity with poly(ADP-ribose) glycohydrolase of *N. gruberi*. HaCPV-5 also has a poly(ADP-ribose) glycohydrolase domain in segment 5 [41]. The genomic DNA of the natural host can sometimes be transferred horizontally into the granulovirus genome by a transposable element, as in an insertion mutant of *Cydia pomonella granulovirus* (CpGV) [42]. Horizontal transfer can also occur between different dsRNA viral species, including mycoviruses [43]. A phylogenetic analysis of UDP-glucosyltransferases of baculoviruses and their homologues in other insects and animals revealed that the UDP-glucosyltransferase genes of the baculoviruses were transferred from insect hosts [44]. The homology between the predicted protein encoded by S6 and insect proteins may be explained by the occasional horizontal transfer of these genes from insect species during the evolutionary process. Therefore, these six segments of DpCPV-MC (S6, S10, S11, S14, S15, and S16) might have been obtained by horizontal gene transfer from host insects or other organisms present in the host insects. The southern blot hybridization assay confirmed that the six dsRNA segments (S6, S10, S11, S14, S15, and S16) were not acquired from the alternative host *S. exigua* (Figure 6).

Two types of RNA secondary structures, the 5′–3′ panhandle structure and a stem–loop structure, are formed in the 5′- and 3′-untranslated regions of the genome segments of the reoviruses [4]. The 5′–3′ panhandle structure is formed by base-pairing between the 5′ and 3′ ends of the plus-strand RNA terminal sequences, and the stem–loop structure is formed by either the 5′ terminal sequence or the 3′ terminal sequence. The RNA secondary structures and a nonbase-paired 3′ tail play important roles in RNA packaging, replication (minus-strand RNA synthesis), and transcription (plus-strand RNA synthesis) [4]. The predicted RNA folding for DpCPV-MC demonstrated that two genome segments (S11 and S16) do not form typical panhandle structures, but rather stem–loop structures, and that nonbase-paired 3′ tails occur in these segments. Both of them (S11 and S16) are unrelated to any protein in the characterized CPVs. We speculate that all 16 genome segments can undergo replication and correct packaging, but that the absence of a typical panhandle in two of the segments (S11 and S16) might cause their transcription to be less efficient than that of the other segments. This phenomenon requires further research.

CPV viral particles have a single capsid shell with 12 surface turrets and a transcriptase complex under each turret, which contains only one specific genome segment. It has been proposed that the genomes of CPVs consist of a maximum of 12 dsRNA segments [4]. Several extra genome segments that are deletion mutants or duplication mutants of the original genome segments have been identified in other CPVs. The genome of HaCPV-14 is composed of 10 dsRNA segments. Four deletion mutants of S5, with differently deleted internal regions, have been identified in HaCPV-14 [45]. The genome of AmCPV-4 is composed of 11 dsRNA segments, and two defective variant forms of S10 were identified in AmCPV-4 [15]. The genome of BmCPV-1 is composed of 10 dsRNA segments. An extra small segment identified in BmCPV-1 was shown to be a deletion mutant of S10, and the extra lane in gel was also considered to be a mixture of deletion mutants of S1, S2, and S3 [46]. These extra segments do not encode a functional protein, but are stable after multiple passages in insects. In all these three cases, the total number of genome segments exceeds 12. The conserved terminal sequences in all these extra segments ensure that they can be correctly packaged into viral particles.

Based on the analysis above, there are 16 segments in the genome of DpCPV-MC, but they can not be packaged into one virion. Since the predicted proteins encoded by three segments (S6, S10 and S14) are related to insect proteins or other viral proteins, the predicted proteins encoded by three segments (S11, S15, and S16) have no obvious sequence similarity to any known protein, even two of them (S11 and S16) transcript less efficiently than the other segments based on RNA secondary structures analysis. We could imply that DpCPV-MC is composed of several genotypes, the ten segments that are related to proteins of other characterized CPVs might constantly exist in all the genotypes, and one or two of the six CPV-unrelated segments co-exist with the ten CPV-related segmets in one DpCPV-MC genotype, thus each virion contains no more than 12 segments. The definite assignments of the 16 segments in different genotypes are not clear based on our present results.

DpCPV-MC was firstly isolated from the mixture with DpCPV-1 from *D. punctatus*, there are 16 dsRNA segments in its genome. As a novel cypovirus, DpCPV-MC will help to better investigate the mechanism of embedding dsRNA segments into virions in the future.
